# Association of intratumoral CD68^+^CD163^+^ M2-like macrophages with survival in metastatic colorectal cancer treated with chemotherapy plus bevacizumab

**DOI:** 10.3389/fimmu.2026.1845691

**Published:** 2026-07-17

**Authors:** Xiaobin Xin, Huixian Qiu, Yan Zang, Lei Zhou, Tao Qin, Qingnuan Kong

**Affiliations:** 1 School of Clinical Medicine, Shandong Second Medical University, Weifang, China; 2 Department of Pathology, Qingdao Hospital, University of Health and Rehabilitation Sciences (Qingdao Municipal Hospital), Qingdao, China; 3 Qingdao Medical College, Qingdao University, Qingdao, China; 4 Department of Oncology, Qingdao Hospital, University of Health and Rehabilitation Sciences (Qingdao Municipal Hospital), Qingdao, China

**Keywords:** bevacizumab resistance, M2-like macrophages, metastatic colorectal cancer, multiplex immunofluorescence, PD-L1, prognosis, tumor-associated macrophages

## Abstract

**Background:**

Metastatic colorectal cancer (mCRC) is a common and highly lethal gastrointestinal malignancy. Although chemotherapy combined with bevacizumab is a standard first-line treatment, post-treatment resistance severely limits patients’ long-term survival. While the remodeling of tumor-associated macrophages (TAMs) is closely related to targeted therapy resistance, the specific impact of TAMs and their programmed death-ligand 1 (PD-L1) expression on post-treatment resistance and prognosis remains unclear. This study aimed to investigate the spatial distribution and immunophenotypic characteristics of CD68^+^CD163^+^ M2-like TAMs and their association with post-treatment resistance and survival.

**Methods:**

We retrospectively analyzed clinical data and tumor tissues from 44 patients with mCRC who received first-line chemotherapy plus bevacizumab and were categorized into resistant and non-resistant groups. Multiplex immunofluorescence and digital image-based quantitative analysis were used to evaluate macrophage markers (CD68 and CD163) and PD-L1 expression in the whole tumor section, tumor areas, and stromal areas.

**Results:**

Compared with the non-resistant group, resistant patients showed significantly increased proportions and densities of CD68^+^CD163^+^ M2-like TAMs and CD68^+^CD163^+^PD-L1^+^ M2-like TAMs in the tumor areas (all p < 0.05). The infiltration levels of these macrophage subsets were significantly associated with post-treatment resistance, but not with conventional clinicopathological features such as sex, age, or tumor stage. Survival analysis showed that a high density of intratumoral CD68^+^CD163^+^ M2-like TAMs was significantly associated with shorter overall survival (OS) (p < 0.05) and showed a trend toward shorter progression-free survival (PFS) (p = 0.06). Multivariate Cox regression further demonstrated that dense intratumoral infiltration of CD68^+^CD163^+^ M2-like TAMs was independently associated with unfavorable OS and PFS.

**Conclusion:**

In mCRC patients exhibiting resistance to chemotherapy combined with bevacizumab, the infiltration of intratumoral CD68^+^CD163^+^ M2-like TAMs and their PD-L1-expressing subsets is significantly elevated. Notably, a high density of intratumoral CD68^+^CD163^+^ M2-like TAMs serves as an independent poor prognostic marker for both PFS and OS. These findings demonstrate that the spatial enrichment of CD68^+^CD163^+^ M2-like TAMs within the tumor microenvironment is closely associated with treatment resistance, suggesting that macrophage-targeted interventions may represent a potential strategy to enhance the efficacy of anti-tumor therapies in mCRC.

## 1 Introduction

Colorectal cancer is one of the most common malignancies worldwide, ranking third in incidence and second in cancer-related mortality (1). According to the National Cancer Center of China, colorectal cancer ranks second in incidence and fourth in mortality among all cancers in China (2). Because early-stage colorectal cancer often presents without obvious clinical symptoms, approximately 60% of patients are diagnosed at an advanced or metastatic stage (3). For these patients, surgical resection alone is usually insufficient, necessitating systemic combinations of chemotherapy and targeted therapies to improve survival. Bevacizumab, the first anti-angiogenic agent approved for metastatic colorectal cancer (mCRC), binds vascular endothelial growth factor A (VEGF-A) and inhibits VEGF-mediated signaling, thereby suppressing tumor angiogenesis and promoting vascular normalization (4). Bevacizumab combined with standard chemotherapy, such as FOLFOX or FOLFIRI, has become a standard first-line treatment for advanced mCRC and can prolong progression-free survival (PFS) and overall survival (OS) (5, 6). However, most patients inevitably develop post-treatment resistance after a certain period of treatment, leading to reduced therapeutic efficacy and tumor progression (7). The potential mechanisms underlying resistance to bevacizumab are extremely complex. In addition to intrinsic molecular alterations in tumor cells, remodeling of the tumor microenvironment (TME) plays a critical role (8). For instance, extracellular matrix remodeling (9) and chronic inflammation (10) can mediate resistance to bevacizumab in mCRC and are associated with patient prognosis. Recent studies have indicated that infiltration of M2 macrophages is correlated with shorter OS in mCRC (11). Nevertheless, reliable biomarkers for predicting resistance to bevacizumab-based combination therapies are still lacking, representing a significant unmet need in the field of precision oncology.

Tumor-associated macrophages (TAMs) are among the most abundant immune cell populations in the TME and promote tumor progression through effects on angiogenesis, immunosuppression, and metabolism (12). Based on their functional states, TAMs are broadly classified into classically activated M1 and alternatively activated M2 phenotypes. Briefly, M1 macrophages are typically induced by lipopolysaccharide or interferon-γ, characterized by high expression of inducible nitric oxide synthase, interleukin-12 (IL-12), and tumor necrosis factor-α, and are endowed with potent antigen-presenting capacity, microbicidal activity, and antitumor functions. Conversely, M2 macrophages are driven by IL-4, IL-13, or IL-10, exhibiting elevated levels of arginase-1, CD206, CD163, and IL-10, and are primarily involved in tissue remodeling, angiogenesis, and immunosuppression, thereby generally exerting pro-tumoral effects in most solid malignancies (13, 14). In most solid tumors, including mCRC, TAMs predominantly exhibit an M2 phenotype, characterized by high expression of markers such as CD163, CD206, and CD204 (13, 15). Recent studies have indicated that M2 TAMs can promote angiogenesis and immune evasion, and are deeply involved in post-treatment resistance to anti-angiogenic therapies. For instance, in bevacizumab-resistant glioblastoma models, reduced macrophage migration inhibitory factor drives macrophage polarization from M1 to M2, and the increased abundance of M2 TAMs correlates with activation of regenerative vascular pathways upon VEGF inhibition, thereby conferring resistance to bevacizumab (16). Additionally, bevacizumab Fc fragment synergizes with Toll−like receptor 4 ligands to polarize macrophages toward an M2b phenotype, and the tumor necrosis factor−α secretion by these cells promotes immunosuppression, metastasis, angiogenesis, and bevacizumab resistance (17). Clinical evidence further indicates that TAM infiltration and tumor neuroendocrine differentiation collectively reduce the efficacy of bevacizumab combined with chemotherapy in patients with advanced mCRC, with TAMs level being the independent prognostic factor for OS (11, 18).Furthermore, the role of programmed cell death 1 (PD-1) and its ligand PD-L1 in tumor immune evasion has been extensively validated (19). While PD-L1 expressed by tumor cells induces effector T cell exhaustion, recent evidence demonstrates that TAMs can also express high levels of PD-L1—sometimes exceeding those of tumor cells—and that this expression increases with macrophage residence time in the tumor (20, 21). PD-L1 expressed on TAMs not only sustains an immunosuppressive microenvironment but may also contribute to resistance to chemotherapy and targeted therapy (22, 23). However, whether the spatial distribution of M2 TAMs within the TME contributes to the development of bevacizumab resistance in mCRC patients remains to be elucidated.

Therefore, we hypothesized that the spatial enrichment of CD68^+^CD163^+^ M2-like TAMs and their co-expression of PD-L1 are critical drivers of post-treatment resistance in mCRC. To accurately evaluate this complex spatial interaction, traditional immunohistochemistry is insufficient. With the advancement of multiplex immunofluorescence (mIF) technologies, researchers are now able to analyze the *in situ* expression and spatial topological relationships of multiple immune markers within the same tissue sample, thereby precisely revealing the interaction patterns among cells within the TME (24). This technology provides a powerful tool for quantitatively evaluating the relationship between CD68^+^CD163^+^ M2-like TAMs, their PD-L1 expression, and resistance to bevacizumab-based combination therapies. Taken together, CD68^+^CD163^+^ M2-like TAMs and their PD-L1 expression may play a key role in the mechanisms of post-treatment resistance to chemotherapy combined with bevacizumab; however, their clinical significance in mCRC patients with bevacizumab resistance remains to be fully elucidated. This study aimed to utilize mIF technology to analyze pretreatment formalin-fixed paraffin-embedded (FFPE) specimens from mCRC patients who exhibited resistance versus non-resistance following treatment with bevacizumab plus chemotherapy. By systematically evaluating the infiltration characteristics of CD68^+^CD163^+^ M2-like TAMs and the differential expression of PD-L1, we aimed to explore their potential clinical value as predictive biomarkers for resistance and prognosis.

## 2 Materials and methods

### 2.1 Patients and clinical data

Clinical and pathological data of mCRC patients treated at Qingdao Municipal Hospital between January 2020 and February 2025 were retrospectively collected through the electronic medical record system. Based on predefined inclusion and exclusion criteria, a total of 44 patients were enrolled in this study. Inclusion criteria were as follows: (1) patients who underwent radical or palliative surgery for primary colorectal cancer, with histopathological confirmation; (2) availability of sufficient FFPE tumor tissue for pathological evaluation; (3) complete clinical and follow-up data, with an expected survival of > 3 months; (4) no contraindications to chemotherapy or bevacizumab; (5) indications for bevacizumab according to the Chinese Society of Clinical Oncology (CSCO) guidelines for mCRC, receiving standard chemotherapy regimens (such as irinotecan, oxaliplatin, or 5-fluorouracil, among others) combined with bevacizumab; and (6) age ≥ 18 years. Exclusion criteria included: (1) malignancies secondary to other types of cancer; (2) concurrent severe cardiovascular, cerebrovascular, hematological, or other severe systemic diseases; (3) hypersensitivity to chemotherapeutic agents; (4) no prior use of bevacizumab or use of bevacizumab after multi-line treatments; (5) incomplete clinical or follow-up survival data; (6) inadequate volume of FFPE tissue blocks or poorly preserved specimens; and (7) pregnant or lactating women. Baseline patient characteristics, including name, gender, age, surgery date, and basic chemotherapy regimens, were recorded. Pathological data included tumor stage, primary tumor location, distant metastasis, mismatch repair (MMR) protein status (MLH1, PMS2, MSH2, MSH6), and epidermal growth factor receptor (EGFR) status, metastatic timing, extent of disease, liver recurrence status, liver recurrence pattern, chemotherapy backbone, molecular covariates, number of treatment cycles, follow-up modifying the treatment regimen (detailed in **Table 1**). The time interval between primary tumor diagnosis and the onset of metastatic disease was stratified using a cutoff of 6 months to define synchronous versus metachronous metastases. Most tumor metastases were detected by imaging (CT/MR), while a small proportion were confirmed by pathological diagnosis following surgical resection. Follow-up was conducted via outpatient visits, documenting treatment regimens, examination results, and survival status. Treatment efficacy was evaluated according to the Response Evaluation Criteria in Solid Tumors (RECIST) version 1.1 (25). The resistant group was defined as patients who experienced disease progression (PD: ≥ 20% increase in the sum of target lesion diameters with an absolute increase of ≥ 5 mm, appearance of new lesions, or progression of non-target lesions) within ≤6 cycles of chemotherapy combined with bevacizumab. The non-resistant group was defined as patients who experienced disease progression after >6 cycles or had no disease progression, including those who achieved complete response (CR: disappearance of all target lesions and no new lesions), partial response (PR: ≥30% decrease in the sum of target lesion diameters), or stable disease (SD: neither sufficient shrinkage to qualify for PR nor sufficient increase to qualify for PD). This study was approved by the Ethics Committee of Qingdao Municipal Hospital (Ethics No.: XS202310018) and complied with international ethical standards. All FFPE tissue blocks were provided by the Department of Pathology of the hospital.

**Table 1:** Baseline clinicopathological characteristics of mCRC patients.

Characteristics		Total (n=44)	Cohort information		*p* value
			Resistant group (n=22)	Non-resistant group (n=22)	
Gender					0.361
	Male	19 (43.1%)	11 (50.0%)	8 (36.3%)	
	Female	25 (56.9%)	11 (50.0%)	14 (63.6%)	
Age (years), n (%)					0.365
	>60	23 (52.3%)	13 (59.1%)	10 (45.5%)	
	≤60	21 (47.7%)	9 (40.9%)	12 (54.5%)	
T stage, n (%)					0.410
	3	7 (15.9%)	2 (9.1%)	5 (22.7%)	
	4	37 (84.1%)	20 (90.9%)	17 (77.2%)	
N stage, n (%)					0.799
	0	8 (18.1%)	4 (18.1%)	4 (18.1%)	
	1	20 (45.4%)	9 (40.9%)	11 (50.0%)	
	2	16 (36.3%)	9 (40.9%)	7 (31.8%)	
Distant metastasis, n (%)					0.948
	None	8 (18.1%)	4 (18.1%)	4 (18.1%)	
	Lung	17 (38.6%)	9 (40.9%)	8 (36.3%)	
	Liver	21 (47.7%)	12 (54.5%)	9 (40.9%)	
	Peritoneum	5 (11.3%)	2 (9.1%)	3 (13.6%)	
	Bone or brain	3 (6.8%)	2 (9.1%)	1 (4.5%)	
Tumor location, n (%)					0.395
	Ileocecal	4 (9.0%)	2 (9.1%)	2 (9.1%)	
	Ascending colon	9 (20.4%)	6 (27.2%)	3 (13.6%)	
	Transverse colon	3 (6.8%)	3 (13.6%)	0 (0%)	
	Descending colon	2 (4.5%)	1 (4.5%)	1 (4.5%)	
	Sigmoid colon	19 (43.1%)	7 (31.8%)	12 (54.5%)	
	Rectum	7 (15.9%)	3 (13.6%)	4 (18.1%)	
pMMR					
	Yes	44 (100%)	22 (100%)	22 (100%)	
	No	0 (0.0%)	0 (0.0%)	0 (0.0%)	
EGFR					0.370
	–	31 (70.4%)	18 (81.8%)	13 (59.0%)	
	±	2 (4.5%)	0 (0.0%)	2 (9.1%)	
	Weak/+	5 (11.3%)	2 (9.1%)	3 (13.6%)	
	+	6 (13.6%)	2 (9.1%)	4 (18.1%)	
Metastatic timing					0.486
	Synchronous	11 (25.0%)	7 (31.8%)	4 (18.2%)	
	Metachronous	33 (75.0%)	15 (68.2%)	18 (81.8%)	
Extent of disease ^a^					0.658
	Liver-only	6/35 (17.1%)	2 (11.8%)	4 (22.2%)	
	Multi-organ	29/35 (82.9%)	15 (88.2%)	14 (77.8%)	
Detection of liver metastases ^b^					0.104
	Imaging	17/21 (81.0%)	8 (66.7%)	9 (100%)	
	Partial hepatectomy	4/21 (19.0%)	4 (33.3%)	0 (0%)	
Liver recurrence status ^c^					0.545
	Yes	7/11 (63.6%)	3 (50.0%)	4 (80.0%)	
	No	4/11 (36.4%)	3 (50.0%)	1 (20.0%)	
Liver recurrence pattern ^d^					0.029
	Post-hepatectomy recurrence	2/7 (28.6%)	0 (0%)	2 (50.0%)	
	Post-MWA recurrence	2/7 (28.6%)	0 (0%)	2 (50.0%)	
	Post-TACE recurrence	3/7 (42.8%)	3 (100%)	0 (0%)	
Chemotherapy backbone					0.837
	FOLFOX-BEV	2 (4.5%)	1 (4.5%)	1 (4.5%)	
	FOLFIRI-BEV	4 (9.1%)	2 (9.1%)	2 (9.1%)	
	CAPOX-BEV	16 (36.4%)	7 (31.8%)	9 (40.9%)	
	IRI-BEV	5 (11.4%)	3 (13.6%)	2 (9.1%)	
	CAP-BEV	5 (11.4%)	2 (9.1%)	3 (13.6%)	
	XELIRI-BEV	2 (4.5%)	2 (9.1%)	0 (0%)	
	SALIRI-BEV	6 (13.6%)	4 (18.2%)	2 (9.1%)	
	Other regimens	4 (9.1%)	1 (4.5%)	3 (13.6%)	
Molecular covariates ^e^					0.175
	KRAS	20/31 (64.5%)	12 (70.6%)	8 (57.1%)	
	NRAS	2/31 (6.5%)	2 (11.8%)	0 (0%)	
	BRAF	0/31 (0%)	0 (0%)	0 (0%)	
	Negative	9/31 (29.0%)	3 (17.6%)	6 (42.9%)	
Number of treatment cycles					0.353
	< 6 cycles	17 (38.6%)	7 (31.8%)	10 (45.5%)	
	≥ 6 cycles	27 (61.4%)	15 (68.2%)	12 (54.5%)	
Follow-up modifying the treatment regimen					<0.0001
	< 6 cycles	24 (54.5%)	19 (86.4%)	5 (22.7%)	
	≥ 6 cycles	20 (45.5%)	3 (13.6%)	17 (77.3%)	

T, tumor; N, node; pMMR, proficient mismatch repair status; MWA, microwave ablation; TACE, transarterial chemoembolization; FOLFOX, Folinic acid- Fluorouracil- Oxaliplatin; BEV, Bevacizumab; FOLFIRI, Folinic acid- Fluorouracil- Irinotecan; CAPOX, Capecitabine- Oxaliplatin; IRI, Irinotecan; CAP, Capecitabine; XELIRI, Capecitabine- Irinotecan; SALIRI, Raltitrexed- Irinotecan.

a Extent of disease was assessed in 35 patients.

b Liver metastases were present in 21 patients.

c Liver recurrence after surgical resection was assessed in 11 patients.

d Liver recurrence pattern was evaluable in 7 patients.

e Molecular testing results were available for 31 patients.

### 2.2 Multiplex immunofluorescence staining

Target tumor areas were identified by an experienced pathologist using hematoxylin and eosin (H&E) stained slides, and the corresponding FFPE tissue blocks were retrieved. Consecutive 3 µm-thick sections were cut using a microtome and mounted on anti-drop glass slides. The slides were baked at 65 °C for 30 min, followed by deparaffinization and graded rehydration. Antigen retrieval was performed using ethylenediaminetetraacetic acid (EDTA) buffer (1×) in a microwave at 100 °C for 20 min. After cooling to room temperature naturally, the slides were washed three times with phosphate-buffered saline with Tween 20 (PBST) (3 min each). Tissue regions were marked with an immunohistochemistry pen, and endogenous peroxidase activity was blocked by incubating with a peroxidase blocking reagent for 10 min at room temperature. Fluorescence staining was performed using the tyramide signal amplification (TSA) multiplex technique. The antibodies used were CD68, CD163, pan−CK, and PD−L1 (all from Abcarta, Suzhou, China; dilution 1:200). The staining order was CD68, CD163, CK, and PD−L1. Each staining cycle included: primary antibody incubation (60 min at 37 °C or overnight at 4 °C), PBST washes, horseradish peroxidase (HRP)-conjugated secondary antibody incubation (30 min at 37 °C), PBST washes, and TSA fluorescent dye visualization (1:200 dilution, 10 min at room temperature in the dark). Multiple stripping and staining cycles were sequentially conducted for different targets. After multiplex staining, nuclei were counterstained with 4’,6-diamidino-2-phenylindole (DAPI) for 10 min at room temperature. The slides were washed with PBST, slightly air-dried, mounted with anti-fade mounting medium, and covered with coverslips. Image acquisition was performed using a High Definition Scanner automated whole-slide imaging system to obtain multi-channel high-resolution images. A spectral library was established using single-stained slides to perform spectral unmixing. Nuclei were identified and segmented based on DAPI signals. Subsequently, quantitative analysis of representative regions of interest (ROI), including tumor areas and stromal areas, was performed using HALO image analysis software. The cell proportion was defined as (number of positive cells/total number of cells) × 100%, and cell density was defined as the number of positive cells per ROI area (µm²).

### 2.3 Survival definitions and statistical analysis

OS was defined as the time from initiation of chemotherapy plus bevacizumab to death from any cause or to loss to follow-up (censored at the last known follow-up). PFS was defined as the time from initiation of chemotherapy plus bevacizumab to disease progression or death from any cause. Statistical analyses were performed using SPSS version 25.0 and GraphPad Prism 9.5. Categorical clinicopathological variables were analyzed using the chi-square test or Fisher’s exact test, as appropriate. The Mann-Whitney U test was employed to compare the expression of CD68^+^CD163^+^ M2-like TAMs, PD-L1^+^ cells, and CD68^+^CD163^+^PD-L1^+^ M2-like TAMs between the resistant and non-resistant groups, and the results were visualized using box plots. Correlations between marker expression and clinicopathological characteristics were assessed using Spearman’s rank correlation coefficient analysis. Receiver operating characteristic (ROC) curves were used to evaluate the predictive value of CD68^+^CD163^+^ M2-like TAMs expression levels. The optimal cut-off value was determined by maximizing the Youden index (sensitivity + specificity - 1), stratifying patients into high-expression and low-expression groups (**Supplementary Figures 1**–**4**; **Supplementary Tables 1**, **2**). Kaplan-Meier survival curves (for both PFS and OS) were plotted, and differences between the groups were compared using the Log-rank test. Univariate and multivariate Cox proportional hazards regression models were applied to identify independent prognostic factors. All statistical tests were two-sided, and p < 0.05 was considered statistically significant.

## 3 Results

### 3.1 Clinical characteristics of the patients

A total of 44 patients with mCRC were enrolled in this study and divided into a resistant group and a non-resistant group based on their response to treatment. The cohort consisted of 19 males (43.1%) and 25 females (56.9%), with an overall median age of 61 years (the median age in the resistant group was 62 years). Distant metastases primarily occurred in the liver (47.7%), followed by the lungs (38.6%). The anatomical distribution of tumors was as follows: sigmoid colon (n = 19, 43.1%), ascending colon (n = 9, 20.4%), rectum (n = 7, 15.9%), ileocecal region (n = 4, 9.0%), transverse colon (n = 3, 6.8%), and descending colon (n = 2, 4.5%). All patients tested positive for MLH1, PMS2, MSH2, and MSH6, indicating proficient mismatch repair (pMMR) status. Furthermore, 31 patients (70.4%) were EGFR-negative. For liver metastases, imaging (CT/MRI) was the primary modality (n = 17, 81.0%) for detection, with a minority (n = 4, 19.0%) confirmed by partial hepatectomy pathology. Thirty-one patients (70.4%) underwent molecular testing in our cohort; among them, 20 patients (64.5%) had KRAS mutations. Other clinical characteristics are summarized in **Table 1**. Among the resistant patients, the majority (18 cases, 81.8%) were classified as primarily refractory/early progression, while a smaller subset (4 cases, 18.2%) exhibited acquired resistance. Regarding the non-resistant patients, 15 cases achieved SD, 4 cases achieved CR, and 3 cases achieved PR (**Supplementary Figure 5**). Except for treatment modification due to disease progression, no significant differences in baseline clinical characteristics were observed between the resistant and non-resistant groups (all p > 0.05).

### 3.2 Differential expression of CD68^+^CD163^+^ M2-like TAMs between resistant and non-resistant groups

**Figure 1:**
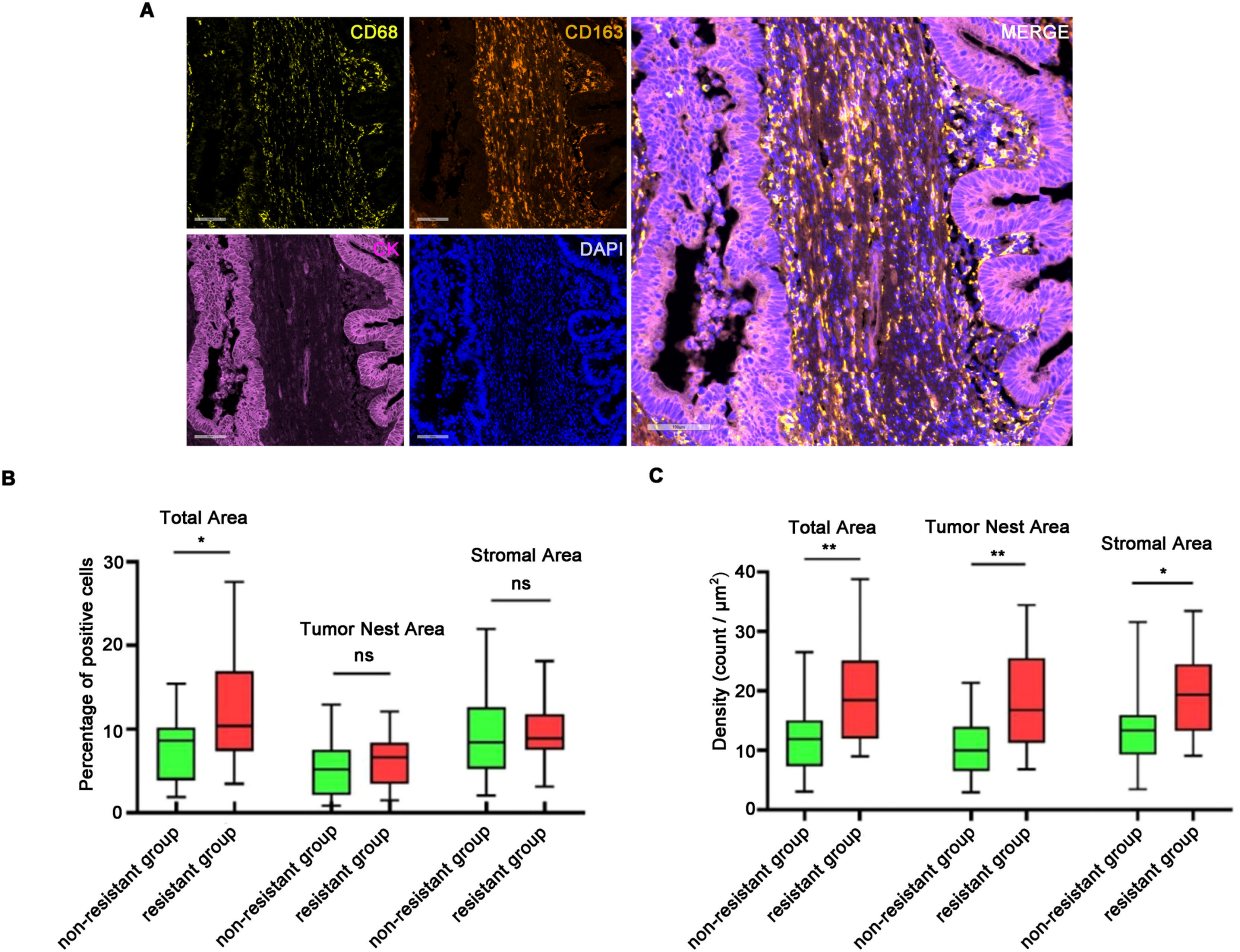
Multiplex immunofluorescence staining of CD68^+^CD163^+^ M2-like TAMs in metastatic colorectal cancer tissues and their expression differences in response to chemotherapy combined with bevacizumab. **(A)** Representative multiplex immunofluorescence (mIF) images of CD68^+^CD163^+^ M2-like TAMs. Scale bar = 100 μm. (Yellow, orange, purple, and blue indicate cells positively expressing CD68, CD163, CK, and DAPI, respectively). **(B)** Comparison of the cell proportions of CD68^+^CD163^+^ M2-like TAMs in the total area, tumor area, and stromal area between the resistant and non-resistant groups in metastatic colorectal cancer patients treated with chemotherapy plus bevacizumab. **(C)** Comparison of the cell densities of CD68^+^CD163^+^ M2-like TAMs in the total area, tumor area, and stromal area between the resistant and non-resistant groups. *p < 0.05, **p < 0.01; ns, not significant (p > 0.05).

To investigate the relationship between the spatial distribution characteristics of the TME at initial diagnosis and the subsequent resistance to chemotherapy combined with bevacizumab, multiplex immunofluorescence was employed for spatial quantitative analysis of immune cell subsets. Based on H&E morphological features, tumor tissues were delineated into tumor areas and stromal areas. The infiltration characteristics—including the distribution proportion and density of CD68^+^, CD163^+^, PD-L1^+^, and CD68^+^CD163^+^PD-L1^+^ M2-like TAMs—were evaluated. Representative mIF images of CD68^+^CD163^+^ M2-like TAMs are shown in **Figure 1A**.

The results demonstrated that in mCRC patients who developed resistance after receiving chemotherapy combined with bevacizumab, the proportion of CD68^+^CD163^+^ M2-like TAMs in the total area was significantly higher than that in the non-resistant group (p < 0.05) (**Figure 1B**). However, spatial compartmental analysis revealed no significant difference in the proportion of TAMs between the two groups within either the tumor areas or the stromal areas (p > 0.05) (**Figure 1B**). These findings suggest that while CD68^+^CD163^+^ M2-like TAMs exhibit a highly immunosuppressive signature across the overall TME, their regional distribution remains relatively homogeneous. The substantial enrichment, high proportion, and increased density of CD68^+^CD163^+^ M2-like TAMs in the total area of patients with resistant mCRC may indicate a remodeling of the immune microenvironment.

### 3.3 Correlation between spatial distribution of CD68^+^CD163^+^ M2-like TAMs in the TME and clinicopathological features

To elucidate the relationship between the spatial distribution of CD68^+^CD163^+^ M2-like TAMs and clinicopathological features in mCRC patients, a correlation analysis was performed using the density and proportion of CD68^+^CD163^+^ M2-like TAMs quantified via mIF. As shown in **Table 2**, the cell density of CD68^+^CD163^+^ M2-like TAMs in the total area, tumor areas, and stromal areas significantly correlated with primary tumor location (p = 0.022, p = 0.028, and p = 0.042, respectively). However, no significant associations were found between CD68^+^CD163^+^ M2-like TAMs density and gender, age, tumor (T) stage, node (N) stage, EGFR status, presence of distant metastasis, or metastasis site (p > 0.05).

**Table 2:** Correlation between CD68^+^CD163^+^ M2-like TAMs infiltration and clinicopathological features in mCRC.

	Total CD68^+^CD163^+^ M2-like TAMs density		Intratumoral CD68^+^CD163^+^ M2-like TAMs density		Stromal CD68^+^CD163^+^ M2-like TAMs density		Total CD68^+^CD163^+^ M2-like TAMs proportion	
	ρ	*p*-value	ρ	*p*-value	ρ	*p*-value	ρ	*p*-value
Gender	-0.164	0.286	-0.157	0.308	-0.132	0.393	0.031	0.843
Age	0.113	0.466	0.099	0.525	0.070	0.652	0.167	0.280
T stage	-0.056	0.717	-0.071	0.647	-0.051	0.740	-0.193	0.209
N stage	-0.168	0.276	-0.174	0.259	-0.104	0.501	-0.112	0.468
Tumor location	0.345	0.022	0.331	0.028	0.307	0.042	0.233	0.129
EGFR	-0.064	0.679	-0.097	0.546	-0.043	0.781	0.151	0.329
Metastasis								
None	-0.027	0.862	0.022	0.887	-0.100	0.517	-0.076	0.625
Lung	-0.069	0.656	-0.033	0.833	-0.146	0.346	-0.058	0.707
Liver	-0.138	0.372	-0.152	0.324	-0.088	0.571	-0.048	0.755
Peritoneum	-0.115	0.458	-0.110	0.479	-0.110	0.479	-0.198	0.197
Bone or brain	-0.025	0.873	0.018	0.909	-0.032	0.837	-0.096	0.536
Drug resistance status	0.426	0.004	0.447	0.002	0.362	0.016	0.336	0.026

TAMs, Tumor-Associated Macrophages; ρ, Spearman’s rank correlation coefficient; T, tumor; N, node; EGFR, epidermal growth factor receptor.

Further analysis indicated that the proportion of CD68^+^CD163^+^ M2-like TAMs in the total area was also not significantly associated with gender, age, T stage, N stage, tumor location, EGFR status, or metastatic status (p > 0.05), suggesting that the total proportion of CD68^+^CD163^+^ M2-like TAMs is largely unaffected by these clinical variables. Notably, both the cell density and proportion of CD68^+^CD163^+^ M2-like TAMs were closely associated with drug resistance (all p < 0.05). The density of CD68^+^CD163^+^ M2-like TAMs in the total area, tumor areas, and stromal areas was significantly higher in the resistant group than in the non-resistant group (p < 0.05) (**Figure 1C**). This indicates that CD68^+^CD163^+^ M2-like TAMs exhibit pronounced enrichment and spatial aggregation in treatment-resistant metastatic mCRC, and their high infiltration levels might play a role in facilitating tumor progression and immunosuppressive effects during the development of drug resistance.

### 3.4 Differential expression analysis of CD68^+^CD163^+^PD-L1^+^ M2-like TAMs

**Figure 2:**
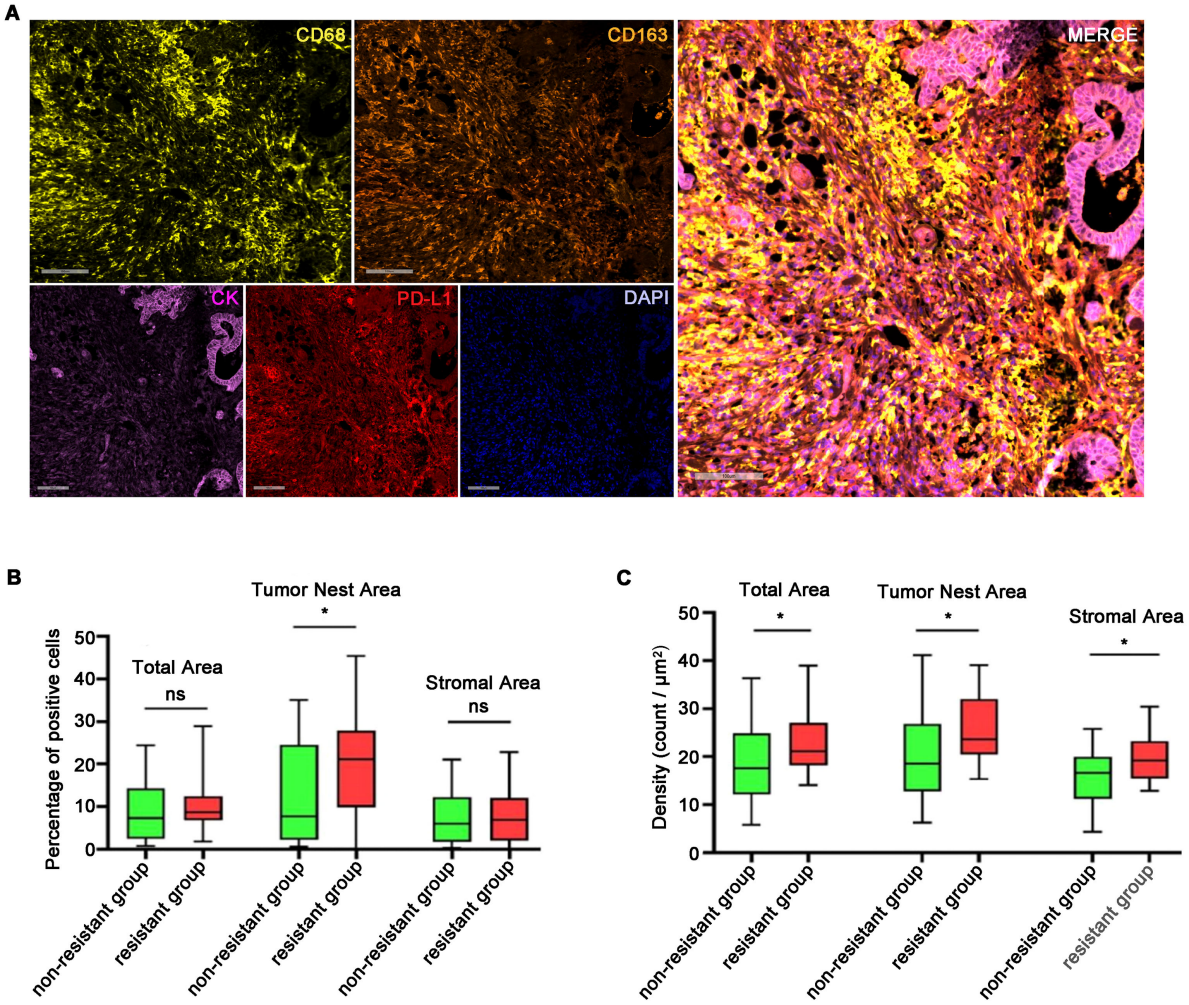
Multiplex immunofluorescence staining and spatial distribution of CD68^+^CD163^+^PD-L1^+^ M2-like TAMs in the metastatic colorectal cancer tumor microenvironment. **(A)** Representative mIF images of CD68^+^CD163^+^ M2-like TAMs and all PD-L1^+^ cells. Scale bars: 100 μm. The colors indicate positive cells expressing specific markers in the tissue: yellow (CD68), orange (CD163), red (PD-L1), purple (CK), and blue (DAPI for nuclei). **(B)** Comparison of the proportions of CD68^+^CD163^+^PD-L1^+^ M2-like TAMs in the total area, tumor area, and stromal area between the resistant and non-resistant groups in metastatic colorectal cancer patients treated with chemotherapy combined with bevacizumab. **(C)** Differences in the cell density of CD68^+^CD163^+^PD-L1^+^ M2-like TAMs in the total area, tumor area, and stromal area between the resistant and non-resistant groups. *p < 0.05; ns, not significant, p > 0.05.

A comparative analysis was conducted on the spatial distribution of CD68^+^CD163^+^PD-L1^+^ M2-like TAMs in mCRC tissues from the resistant and non-resistant groups. Quantitative mIF analysis revealed that the proportion and density of CD68^+^CD163^+^PD-L1^+^ M2-like TAMs in the tumor areas was significantly higher in the resistant group than in the non-resistant group (p < 0.05) (**Figure 2**; **Supplementary Figures 6**, **7**). This finding may imply that the activation of immunosuppressive macrophages and the PD−L1 signaling axis could be involved, at least in part, in regulating the immune microenvironment of bevacizumab−resistant tumors, although further investigation is needed. Further spatial compartmentalization analysis showed no statistically significant difference in the proportion of CD68^+^CD163^+^PD-L1^+^ M2-like TAMs between the two groups within the total area and stromal areas (p > 0.05) (**Figure 2**), implying a relatively stable overall distribution of this cellular subset. Overall, CD68^+^CD163^+^PD-L1^+^ M2-like TAMs demonstrated a trend of tumor enrichment in resistant mCRC, but their overall distribution remains relatively stable, which may be attributed to a highly generalized immunosuppressive state throughout the tumor.

### 3.5 Correlation between CD68^+^CD163^+^PD-L1^+^ M2-like TAMs and clinicopathological features

To further explore the potential link between immunosuppressive cell subsets within the TME and resistance to combined chemotherapy and bevacizumab, the infiltration patterns of CD68^+^CD163^+^PD-L1^+^ M2-like TAMs and their correlations with clinicopathological features were systematically analyzed. The results revealed that most of the differences were not statistically significant in the distribution of CD68^+^CD163^+^PD-L1^+^ M2-like TAMs across various clinicopathological factors, including gender, age, N stage, EGFR status, tumor location, presence of distant metastasis (excluding lung), and metastasis site (non-lung) (**Table 3**). This indicates that the overall infiltration of this subset is not overtly correlated with routine clinical variables.

**Table 3:** Infiltration intensity of CD68^+^CD163^+^PD-L1^+^ M2-like TAMs in mCRC and its specific associations with clinicopathological features.

	Total CD68^+^CD163^+^PD-L1^+^ M2-like TAMs density		Intratumoral CD68^+^CD163^+^PD-L1^+^ M2-like TAMs density		Stromal CD68^+^CD163^+^PD-L1^+^ M2-like TAMs density		Intratumoral CD68^+^CD163^+^PD-L1^+^ M2-like TAMs proportion	
	ρ	*p*-value	ρ	*p*-value	ρ	*p*-value	ρ	*p*-value
Gender	-0.269	0.077	-0.280	0.066	-0.132	0.393	-0.150	0.331
Age	0.091	0.555	0.113	0.466	0.084	0.587	0.217	0.158
T stage	0.081	0.602	0.095	0.538	0.086	0.580	0.321	0.034
N stage	0.070	0.653	0.013	0.936	0.125	0.417	0.215	0.161
Tumor location	0.103	0.506	0.158	0.304	0.123	0.427	0.110	0.477
EGFR	0.000	0.998	-0.012	0.940	0.138	0.371	0.263	0.084
Metastasis								
None	-0.105	0.497	-0.091	0.559	-0.139	0.367	-0.237	0.121
Lung	-0.269	0.077	-0.226	0.141	-0.342	0.023	-0.098	0.526
Liver	0.034	0.826	-0.056	0.720	0.056	0.720	-0.253	0.098
Peritoneum	0.214	0.163	0.151	0.327	0.276	0.069	-0.010	0.946
Bone or brain	-0.039	0.801	0.018	0.909	0.011	0.945	-0.103	0.506
Drug resistance status	0.315	0.037	0.376	0.012	0.333	0.027	0.319	0.035

PD-L1, Programmed Cell Death Ligand 1; TAMs, Tumor-Associated Macrophages; ρ, Spearman’s rank correlation coefficient; T, tumor; N, node; EGFR, epidermal growth factor receptor.

Importantly, a significant association was identified between the density and proportion of CD68^+^CD163^+^PD-L1^+^ M2-like TAMs and resistance to combined bevacizumab treatment (all p < 0.05) (**Table 3**). The infiltration level of CD68^+^CD163^+^PD-L1^+^ M2-like TAMs was markedly higher in the resistant group, suggesting the involvement of the PD-L1 signaling axis in remodeling the bevacizumab-associated tumor immune microenvironment. The expression of PD-L1 may confer additional immunosuppressive effects on CD68^+^CD163^+^ M2-like TAMs, enabling tumor cells to evade immune surveillance and thereby decreasing sensitivity to anti-angiogenic therapy.

### 3.6 Prognostic impact of CD68^+^CD163^+^ M2-like TAMs in the overall mCRC cohort

**Figure 3:**
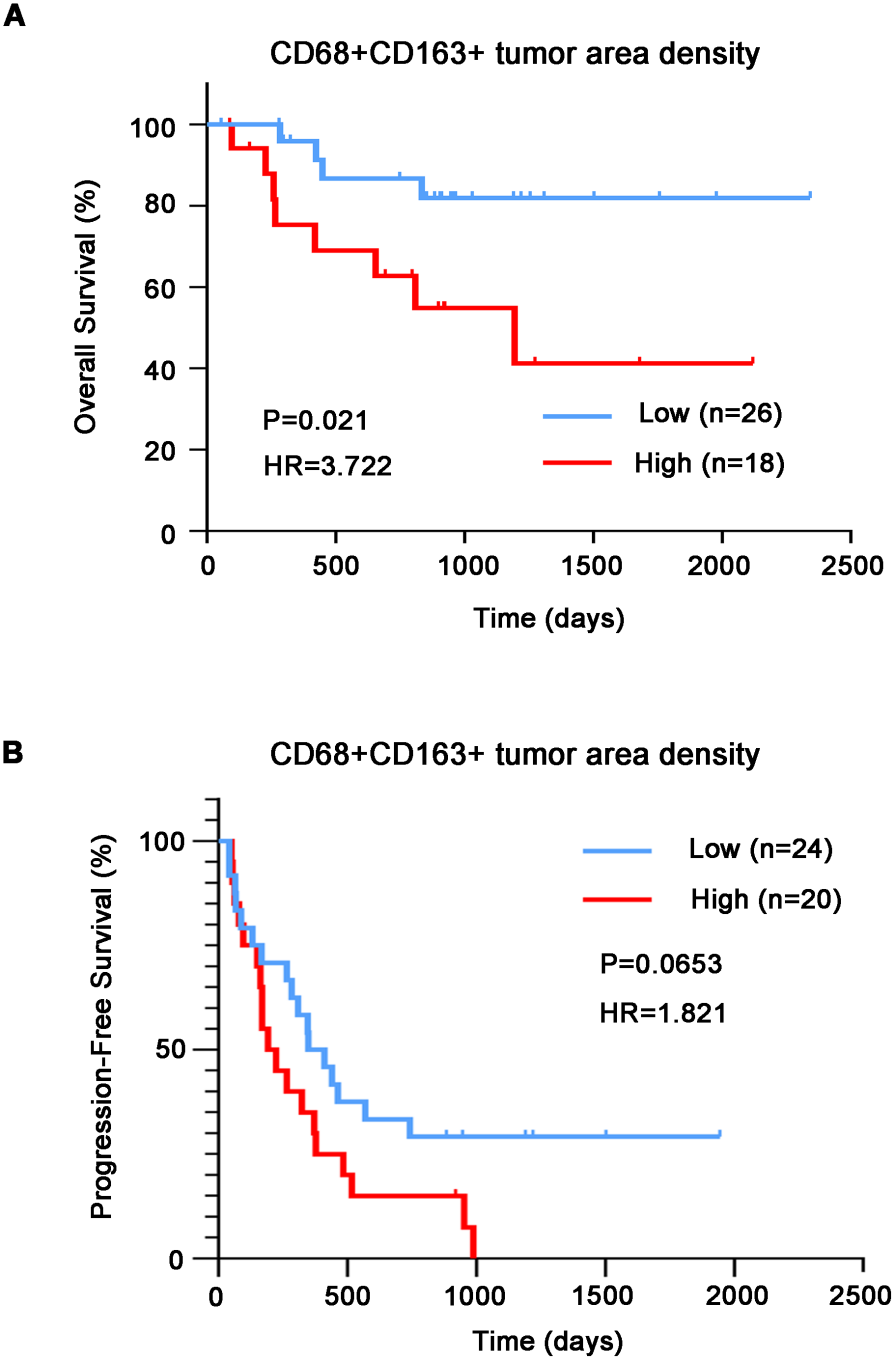
Survival analysis based on CD68^+^CD163^+^ M2-like TAM density in the overall metastatic colorectal cancer population. Panel **(A)** shows a Kaplan-Meier survival curve comparing overall survival for patients with low (blue, n=26) and high (red, n=18) CD68^+^CD163^+^ tumor area density, with high density associated with reduced survival (P=0.021, HR=3.722). Panel **(B)** shows a Kaplan-Meier curve comparing progression-free survival for patients with low (blue, n=24) and high (red, n=20) CD68^+^CD163^+^ tumor area density, where high density shows a trend toward worse outcomes (P=0.0653, HR=1.821).

To investigate the prognostic value of CD68^+^CD163^+^ M2-like TAMs infiltration in mCRC patients, the cohort was divided into high-density and low-density groups based on the mIF-derived cell density of CD68^+^CD163^+^ M2-like TAMs in the total area and tumor areas. As illustrated in **Figure 3**, patients with high CD68^+^CD163^+^ M2-like TAMs density in the tumor areas had a significantly shorter OS than those with low density (HR = 3.722, p = 0.021). However, this difference was not statistically significant when evaluated based on density in the total area. Regarding PFS in the tumor areas, the difference did not reach statistical significance (HR = 1.821, p = 0.065); however, patients with high CD68^+^CD163^+^ M2-like TAMs density exhibited a trend toward shorter PFS.

Univariate Cox regression analysis revealed that both the number of organ metastases and the density of CD68^+^CD163^+^ M2-like TAMs in the tumor areas were significantly associated with OS (p < 0.05). Subsequent multivariate analysis confirmed that CD68^+^CD163^+^ M2-like TAMs density in the tumor areas remained an independent poor prognostic factor for OS in mCRC patients (HR = 5.944, 95% CI: 1.492–23.683, p = 0.012). Analyses for PFS yielded similar results. Univariate analysis showed that multiple metastatic organs (≥2), along with high levels of CD68^+^CD163^+^ M2-like TAMs, total CD68^+^CD163^+^ M2-like TAMs average cellular intensity, intratumoral CD68^+^CD163^+^ M2-like TAMs average cellular intensity, and CD68^+^CD163^+^PD-L1^+^ M2-like TAMs percentage, were significantly correlated with PFS (P < 0.05). In the multivariate model, CD68^+^CD163^+^ M2-like TAMs density in the tumor areas persisted as an independent predictor of poor PFS (HR = 2.129, 95% CI: 1.059–4.278, p = 0.034) (**Tables 4**, **5**). Taken together, these findings indicate that high CD68^+^CD163^+^ M2-like TAMs infiltration is not only closely linked to shortened OS but also adversely affects PFS, underscoring its potential value as a prognostic biomarker.

**Table 4:** Univariable and multivariable analysis for PFS.

	Univariate analysis			Multivariate analysis		
	Hazard ratio	95% CI	*p*-value	Hazard ratio	95% CI	*p*-value
Tumor location (transverse colon)	3.673	0.800-16.867	0.094			
Tumor differentiation (poorly)	1.216	0.571-2.590	0.612			
T stage (T4)	2.098	0.739-5.954	0.164			
N stage (N2)	1.270	0.793-2.033	0.319			
Metastasis (present)	1.099	0.457-2.642	0.834			
TNM stage (IV)	1.847	0.439-7.769	0.403			
Number of metastatic organs (≥2)	2.452	1.146-5.246	0.021	2.932	1.299-6.620	0.010
Lung metastasis (present)	1.268	0.655-2.455	0.481			
Liver metastasis (present)	1.644	0.851-3.177	0.139			
Peritoneal metastasis (present)	1.025	0.396-2.654	0.959			
Bone or brain metastasis (present)	0.864	0.207-3.611	0.841			
EGFR (+)	0.844	0.616-1.155	0.289			
Total CD68^+^CD163^+^ M2-like TAMs proportion (high)	0.348	0.123-0.989	0.048	0.566	0.153-2.094	0.394
Total CD68^+^CD163^+^ M2-like TAMs density (high)	0.403	0.182-0.889	0.024	0.633	0.255-1.570	0.324
Intratumoral CD68^+^CD163^+^ M2-like TAMs density (high)	1.846	0.952-3.578	0.070	2.129	1.059-4.278	0.034
Stromal CD68^+^CD163^+^ M2-like TAMs density (high)	0.650	0.314-1.344	0.245			
Total CD68^+^CD163^+^PD-L1^+^ M2-like TAMs proportion (high)	0.498	0.257-0.967	0.040	0.529	0.253-1.104	0.090
Intratumoral CD68^+^CD163^+^PD-L1^+^ M2-like TAMs proportion (high)	1.007	0.522-1.942	0.983			
Total CD68^+^CD163^+^PD-L1^+^ M2-like TAMs density (high)	0.845	0.433-1.650	0.622			
Intratumoral CD68^+^CD163^+^PD-L1^+^ M2-like TAMs density (high)	1.119	0.581-2.156	0.737			
Stromal CD68^+^CD163^+^PD-L1^+^ M2-like TAMs density (high)	1.119	0.581-2.156	0.737			

PFS, progression-free survival; CI, confidence interval; T, tumor; N, node; TNM, tumor-node-metastasis; EGFR, epidermal growth factor receptor; TAMs, Tumor-Associated Macrophages; PD-L1, Programmed Cell Death Ligand 1.

**Table 5:** Univariable and multivariable analysis for OS.

	Univariate analysis			Multivariate analysis		
	Hazard ratio	95% CI	*p*-value	Hazard ratio	95% CI	*p*-value
Age (≤60)	0.661	0.208-2.093	0.481			
Tumor location (ascending colon)	1.491	0.247-8.983	0.663			
Tumor differentiation (poorly)	1.424	0.383-5.291	0.597			
T stage (T4)	27.895	0.049-15741.989	0.303			
N stage (N2)	0.879	0.415-1.861	0.736			
Metastasis (present)	0.642	0.173-2.384	0.508			
TNM stage (IV)	0.367	0.079-1.706	0.201			
Number of metastatic organs (≥2)	3.533	1.011-12.347	0.048	3.339	0.853-13.074	0.083
Lung metastasis (present)	1.207	0.381-3.822	0.749			
Liver metastasis (present)	0.969	0.307-3.058	0.957			
Peritoneal metastasis (present)	0.702	0.090-5.453	0.735			
Bone or brain metastasis (present)	4.275	0.917-19.934	0.064	7.196	1.109-46.675	0.039
EGFR (+)	0.833	0.481-1.442	0.514			
Total CD68^+^CD163^+^ M2-like TAMs proportion (high)	2.533	0.685-9.373	0.164			
Total CD68^+^CD163^+^ M2-like TAMs density (high)	2.846	0.364-22.243	0.319			
Intratumoral CD68^+^CD163^+^ M2-like TAMs density (high)	3.745	1.125-12.468	0.031	5.944	1.492-23.683	0.012
Stromal CD68^+^CD163^+^ M2-like TAMs density (high)	1.432	0.313-6.546	0.643			
Total CD68^+^CD163^+^PD-L1^+^ M2-like TAMs proportion (high)	0.505	0.159-1.606	0.247			
Intratumoral CD68^+^CD163^+^PD-L1^+^ M2-like TAMs proportion (high)	1.067	0.343-3.313	0.911			
Total CD68^+^CD163^+^PD-L1^+^ M2-like TAMs density (high)	0.363	0.109-1.210	0.099			
Intratumoral CD68^+^CD163^+^PD-L1^+^ M2-like TAMs density (high)	1.855	0.587-5.864	0.293			
Stromal CD68^+^CD163^+^PD-L1^+^ M2-like TAMs density (high)	1.855	0.587-5.864	0.293			

OS, overall survival; CI, confidence interval; T, tumor; N, node; TNM, tumor-node-metastasis; EGFR, epidermal growth factor receptor; TAMs, Tumor-Associated Macrophages; PD-L1, Programmed Cell Death Ligand 1.

## 4 Discussion

In this study, we used multiplex immunofluorescence and spatial quantitative analysis to examine tissue specimens from 44 patients with advanced mCRC treated with chemotherapy plus bevacizumab. We found that TAMs showed a heterogeneous spatial distribution within the mCRC tumor microenvironment. Compared with the non-resistant group, the resistant group showed significant enrichment of both CD68^+^CD163^+^ M2-like TAMs and CD68^+^CD163^+^PD-L1^+^ M2-like TAMs. Survival analysis indicated that a high density of CD68^+^CD163^+^ M2-like TAMs in the tumor areas was significantly associated with a shorter OS in the total population (p < 0.05), and these patients also showed a trend towards worse PFS (p = 0.065). Furthermore, multivariate Cox regression analysis incorporating clinical characteristics confirmed that a high density of CD68^+^CD163^+^ M2-like TAMs in the tumor areas is an independent poor prognostic factor for both OS and PFS in mCRC patients. This study reveals that high intratumoral M2−like TAMs infiltration may contribute to resistance to chemotherapy combined with anti−angiogenic therapy and may also be associated with unfavorable clinical outcomes.

Tumor initiation, progression, and targeted drug resistance depend not only on the intrinsic biological characteristics of tumor cells but are also intricately linked to TME remodeling. The concept of the local biological tumor environment, first proposed by Ioannides and Whiteside (26), has been extensively validated in recent years, proving its decisive role in mCRC progression and the evolution of drug resistance. Therefore, deeply deciphering the spatial infiltration characteristics of immune cells and the expression of their functional markers within the TME is crucial for evaluating patient prognosis and treatment response. This allows for the precise screening of advantageous populations prior to treatment, providing a theoretical basis for individualized mCRC therapy. Traditional immunohistochemistry has inherent limitations, whereas mIF technology enables the simultaneous *in situ* detection of multiple immune markers on a single tissue section (24). By precisely identifying cell phenotypes and performing spatial quantitative analysis, mIF has emerged as a cutting-edge tool for unraveling the complex network relationships between tumors and the TME (27), and has been widely applied in evaluating the efficacy of immunotherapy (28) and neoadjuvant chemotherapy (17). In the current study, we employed mIF to perform multiplex staining for CD68, CD163, and PD-L1 on patient tissue sections, dissecting the spatial distribution features of TAMs and their PD-L1 expression in the TME, with the aim of identifying potential biomarkers for predicting bevacizumab resistance and prognosis.

In recent years, macrophage biology has achieved major breakthroughs in the field of tumor immunology (29). As one of the most abundant immune cell populations in the TME, TAMs primarily exhibit two polarization states: classically activated M1 (anti-tumor) and alternatively activated M2 (pro-tumor) phenotypes. In the TME of most solid tumors, TAMs generally skew towards M2 polarization (12, 13). M2 TAMs continuously drive tumor growth, invasion, and metastasis by exerting anti-inflammatory and pro-angiogenic effects (30). Because CD163 is highly and specifically expressed on the surface of M2 macrophages, it is frequently utilized as a core marker to distinguish the M2 phenotype (31, 32). Accordingly, we defined and quantified CD68^+^CD163^+^ M2-like TAMs in the TME using CD68^+^CD163^+^ dual positivity. Our results revealed that in patients who developed resistance after receiving chemotherapy plus bevacizumab, the proportion of CD68^+^CD163^+^ M2-like TAMs in the total area was significantly higher than that in the non-resistant group. Concurrently, the cell density of CD68^+^CD163^+^ M2-like TAMs in the resistant group showed significant enrichment across the total area, tumor areas, and stromal (p < 0.05). Clinicopathological correlation analysis further confirmed that the density and proportion of CD68^+^CD163^+^ M2-like TAMs were not significantly associated with routine clinical variables (such as age or stage) but were highly correlated with resistance to anti-tumor therapy. This finding suggests that the inherent immunosuppressive state of the tumor immune microenvironment prior to anti-angiogenic therapy—namely, the spatial expansion and aggregation of CD68^+^CD163^+^ M2-like TAMs—may be a pivotal mechanism driving primary or secondary resistance to chemotherapy combined with bevacizumab.

Importantly, survival analysis demonstrated that CD68^+^CD163^+^ M2-like TAMs hold significant value for predicting long-term prognosis. Kaplan-Meier survival curves revealed that patients with a high density of CD68^+^CD163^+^ M2-like TAMs in the tumor areas had a significantly shorter OS compared to the low-density group (p < 0.05); regarding PFS, the high-density group also displayed a distinct trend towards earlier disease progression (p = 0.065). Notably, after adjusting for clinical confounders, multivariate Cox regression analysis further indicated that a high cell density of CD68^+^CD163^+^ M2-like TAMs in the tumor areas was potentially associated with worse OS (HR = 5.944, p = 0.012) and PFS (HR = 2.129, p = 0.034) in mCRC patients, suggesting a possible independent prognostic role.

The discrepancy between the significant impact on OS and the marginal significance on PFS in the univariate analysis may be explained by several factors. First, compared to PFS, which relies on imaging follow-up and subjective evaluation criteria (RECIST), OS serves as an objective “hard endpoint.” It is unaffected by measurement errors or follow-up intervals, thus more authentically and stably reflecting the long-term clinical value of the biomarker. Second, a patient’s OS consists of PFS and post-progression survival (PPS). The highly immunosuppressive microenvironment mediated by CD68^+^CD163^+^ M2-like TAMs may not only drive treatment resistance in the early stages of the disease but also reflect an intrinsic tumor aggressiveness that severely attenuates the patient’s response to subsequent lines of therapy (i.e., shortens the PPS). This long-term biological effect is more prominently captured by the OS endpoint. Finally, the significant difference in PFS observed in the multivariate analysis (p < 0.05) indicates that, after excluding clinical confounding factors such as the number of metastatic organs, a high density of CD68^+^CD163^+^ M2-like TAMs is indeed a key independent driver of disease progression. Our conclusions are consistent with several previous studies; for instance, Ding et al. (33) and Xue et al. (34) both found that high infiltration of CD163^+^ TAMs is an independent risk factor for shortened survival and poor prognosis in mCRC patients. Our data provide preliminary evidence that, in the tumor areas, high-density CD68^+^CD163^+^ M2-like TAMs may correlate with an immunosuppressive microenvironment, accelerated tumor progression, and compromised benefit from anti−angiogenic therapy. Whether these cells serve as independent drivers of resistance or reliable prognostic indicators requires further investigation.

However, relying solely on CD68^+^CD163^+^ macrophages to define M2 TAMs still has limitations, because the traditional M1/M2 binary classification inadequately captures the functional diversity of TAMs within the tumor microenvironment. Recent single-cell RNA sequencing (scRNA-seq) studies have identified four novel TAM subpopulations in distinct solid tumors based on core gene signatures, including FCN1^+^, SPP1^+^, C1Q^+^, and CCL18^+^ TAMs. Among these, C1Q^+^ and SPP1^+^ are the major subpopulations, while FCN1^+^ and CCL18^+^ are relatively rare but functionally well-defined subpopulations (35). SPP1^+^ macrophages are enriched in hypoxic and necrotic tumor regions and portend worse outcome in colon cancer. In contrast, IL4I1^+^ macrophages phagocytose dying cells in areas with high cell turnover and predict good outcome in colon cancer (36). In our previous study, we performed staining for SPP1, which showed only rare positive signals in our cohort. Therefore, we ruled out the possibility that SPP1^+^ M2−like TAMs represent a major subpopulation in the present study.

Studies have shown that the combination of TAM morphology and CD163 expression intensity can identify biologically distinct macrophage populations. Macrophage morphology is not merely a structural feature, but rather an external manifestation of intrinsic functional heterogeneity, with large TAMs being directly associated with enhanced immunosuppression and extremely poor prognosis (37). Recent research has revealed that TAMs expressing high levels of CD163 are not only a reliable marker of M2 polarization but also a potent immunosuppressive cell subset closely associated with tumor progression and poor prognosis. Many studies have found that CD163^+^ TAMs perform specific pro-tumorigenic functions across multiple solid tumor types, and that the association with poor outcome was more frequently observed when CD163^+^ cells were measured at the tumor periphery compared to more central regions (38, 39). In clear cell renal cell carcinoma, CD163 expression is positively correlated with tumor size, further confirming its ability to promote tumor growth (40). However, whether specific M2−like TAM subsets drive bevacizumab resistance remains poorly understood, and the underlying mechanisms warrant further experimental investigation.

Early synchronous colorectal liver metastasis lesions possess a highly immunosuppressive milieu characterized by large proliferative CTLA4^+^ immunoregulatory TAMs. The presence of a large population of proliferative immunoregulatory TAM subpopulations in liver metastasis — which are highly consistent with the CD163^+^PCNA^+^ phenotype at both functional and phenotypic levels — is significantly associated with poor prognosis (41). The mechanisms by which TAMs promote angiogenesis and therapy resistance include the secretion of molecules such as VEGF and MMPs (42). Deletion of CCL7 in myeloid cells resulted in reduced accumulation of immunosuppressive TAMs and increased infiltration of activated CD8^+^ T cells within the tumor, suggesting that CCL7 may serve as a potential therapeutic target in combination with immune checkpoint inhibitors (43).

PD-L1 is a critical immune checkpoint responsible for maintaining immune tolerance and mediating tumor immune evasion (44). Within the TME, TAMs can express PD-L1 to bind with PD-1 on the surface of CD8^+^ T cells, directly leading to T cell exhaustion and thereby dampening the anti-tumor immune response (45). Furthermore, recent studies have discovered that the PD-1/PD-L1 axis is closely linked to chemoresistance. For example, in gastric cancer and multiple myeloma, PD-L1 signaling can induce tumor cell resistance to chemotherapeutic agents by activating pathways such as PI3K/AKT (46, 47). In this study, we performed spatial quantitative analysis of the co-expression of PD-L1 and CD68^+^CD163^+^ M2-like TAMs using mIF. The results demonstrated that the proportion of CD68^+^CD163^+^PD-L1^+^ M2-like TAMs in the total area of resistant patients was significantly higher than that in the non-resistant group (p < 0.05). Correlation analysis similarly showed that the infiltration level of these cells was independent of routine pathological features but significantly associated with resistance to chemotherapy plus bevacizumab (p = 0.033). These observations suggest that PD-L1 upregulation on CD68^+^CD163^+^ M2-like TAMs may further reinforce the immunosuppressive microenvironment and contribute to treatment resistance. However, because PD-1 expression on T cells was not assessed, any mechanistic inference regarding PD-1/PD-L1-mediated immunosuppression should be interpreted cautiously.

This study has several limitations. First, its retrospective design and relatively small sample size (n = 44) limit statistical power, particularly for subgroup and survival analyses. Second, defining resistance solely by RECIST assessment after six treatment cycles does not fully dissect the dynamic evolution of resistance, nor does it capture delayed responses to therapy. Third, variation in PD-L1 antibody clones and scoring criteria may influence the reproducibility of results. Fourth, the lack of analysis of peritumoral and invasive-margin regions limits evaluation of spatial immune features that may be particularly relevant to anti-angiogenic therapy response. Finally, the molecular mechanisms by which CD68^+^CD163^+^ M2-like TAMs and PD-L1 contribute to bevacizumab resistance were not experimentally validated *in vitro* or *in vivo*. Future studies should include larger multicenter cohorts and mechanistic experiments to validate these findings and assess their clinical applicability.

In conclusion, using multiplex immunofluorescence and spatial quantitative analysis, we demonstrated that CD68^+^CD163^+^ M2-like TAMs and CD68^+^CD163^+^PD-L1^+^ M2-like TAMs are significantly enriched in mCRC tissues from patients resistant to chemotherapy plus bevacizumab. Notably, a high density of intratumoral CD68^+^CD163^+^ M2-like TAMs serves as an independent poor prognostic marker for both PFS and OS. These findings demonstrate that the spatial enrichment of CD68^+^CD163^+^ M2-like TAMs within the tumor microenvironment is closely associated with treatment resistance, suggesting that macrophage-targeted interventions may represent a potential strategy to enhance the efficacy of anti-tumor therapies in mCRC.

## Data Availability

The original contributions presented in the study are included in the article/**Supplementary Material**, further inquiries can be directed to the corresponding author/s.
