# Supplementary material for: Association of intratumoral CD68+CD163+ M2-like macrophages with survival in metastatic colorectal cancer treated with chemotherapy plus bevacizumab
Source: Front Immunol. 2026 Jul 17;17:1845691. doi: 10.3389/fimmu.2026.1845691 (PMC13423976; doi:10.3389/fimmu.2026.1845691)
Supplement: Supplementary file 1 [file DataSheet1.docx]

**Supplementary Information**

**Supplementary Table 1.** Coordinates of the Curve for PFS status.

Cut-off thresholds were determined based on the data shown in this table. Optimal cut‑off: 13.6558; sensitivity: 0.528; specificity: 0.875.

| **Coordinates of the Curve** | | |
| --- | --- | --- |
| Test Result Variable(s): TumorPFS | | |
| Positive if Greater Than or Equal To^a^ | Sensitivity | 1 - Specificity |
| 1.9271 | 1.000 | 1.000 |
| 3.2800 | .972 | 1.000 |
| 3.9013 | .944 | 1.000 |
| 4.3296 | .917 | 1.000 |
| 5.0139 | .889 | 1.000 |
| 6.1749 | .861 | 1.000 |
| 6.8498 | .861 | .875 |
| 6.8947 | .833 | .875 |
| 6.9717 | .833 | .750 |
| 7.1859 | .806 | .750 |
| 8.1359 | .778 | .750 |
| 9.0642 | .750 | .750 |
| 9.3676 | .750 | .625 |
| 9.5606 | .722 | .625 |
| 9.6359 | .694 | .625 |
| 9.9722 | .694 | .500 |
| 10.6923 | .667 | .500 |
| 11.2068 | .639 | .500 |
| 11.4386 | .611 | .500 |
| 11.7926 | .611 | .375 |
| 12.0831 | .583 | .375 |
| 12.3992 | .583 | .250 |
| 13.0390 | .556 | .250 |
| 13.5537 | .528 | .250 |
| 13.6558 | .528 | .125 |
| 14.3203 | .500 | .125 |
| 15.0770 | .472 | .125 |
| 15.6887 | .444 | .125 |
| 16.2139 | .417 | .125 |
| 16.4539 | .389 | .125 |
| 16.8324 | .361 | .125 |
| 17.0519 | .333 | .125 |
| 17.6811 | .306 | .125 |
| 18.5324 | .278 | .125 |
| 18.9521 | .250 | .125 |
| 19.6216 | .222 | .125 |
| 20.7643 | .194 | .125 |
| 22.8297 | .167 | .125 |
| 24.7185 | .167 | .000 |
| 25.8519 | .139 | .000 |
| 27.0657 | .111 | .000 |
| 28.3255 | .083 | .000 |
| 31.7016 | .056 | .000 |
| 34.4118 | .028 | .000 |
| 35.4939 | .000 | .000 |
| a. The smallest cutoff value is the minimum observed test value minus 1, and the largest cutoff value is the maximum observed test value plus 1. All the other cutoff values are the averages of two consecutive ordered observed test values. | | |

**Supplementary Table 2.** Coordinates of the Curve for OS status.

Cut-off thresholds were determined based on the data shown in this table. Optimal cut‑off: 15.0770; sensitivity: 0.667; specificity: 0.687.

| **Coordinates of the Curve** | | |
| --- | --- | --- |
| Test Result Variable(s): TumorOS | | |
| Positive if Greater Than or Equal To^a^ | Sensitivity | 1 - Specificity |
| 1.9271 | 1.000 | 1.000 |
| 3.2800 | 1.000 | .969 |
| 3.9013 | 1.000 | .938 |
| 4.3296 | 1.000 | .906 |
| 5.0139 | 1.000 | .875 |
| 6.1749 | 1.000 | .844 |
| 6.8498 | 1.000 | .813 |
| 6.8947 | .917 | .813 |
| 6.9717 | .917 | .781 |
| 7.1859 | .917 | .750 |
| 8.1359 | .917 | .719 |
| 9.0642 | .917 | .688 |
| 9.3676 | .917 | .656 |
| 9.5606 | .917 | .625 |
| 9.6359 | .917 | .594 |
| 9.9722 | .917 | .563 |
| 10.6923 | .833 | .563 |
| 11.2068 | .750 | .563 |
| 11.4386 | .750 | .531 |
| 11.7926 | .750 | .500 |
| 12.0831 | .750 | .469 |
| 12.3992 | .750 | .438 |
| 13.0390 | .750 | .406 |
| 13.5537 | .667 | .406 |
| 13.6558 | .667 | .375 |
| 14.3203 | .667 | .344 |
| 15.0770 | .667 | .313 |
| 15.6887 | .583 | .313 |
| 16.2139 | .583 | .281 |
| 16.4539 | .500 | .281 |
| 16.8324 | .417 | .281 |
| 17.0519 | .417 | .250 |
| 17.6811 | .417 | .219 |
| 18.5324 | .417 | .188 |
| 18.9521 | .417 | .156 |
| 19.6216 | .417 | .125 |
| 20.7643 | .417 | .094 |
| 22.8297 | .333 | .094 |
| 24.7185 | .333 | .063 |
| 25.8519 | .333 | .031 |
| 27.0657 | .250 | .031 |
| 28.3255 | .167 | .031 |
| 31.7016 | .083 | .031 |
| 34.4118 | .083 | .000 |
| 35.4939 | .000 | .000 |
| a. The smallest cutoff value is the minimum observed test value minus 1, and the largest cutoff value is the maximum observed test value plus 1. All the other cutoff values are the averages of two consecutive ordered observed test values. | | |
