# Supplementary material for: Association of intratumoral CD68+CD163+ M2-like macrophages with survival in metastatic colorectal cancer treated with chemotherapy plus bevacizumab
Source: Front Immunol. 2026 Jul 17;17:1845691. doi: 10.3389/fimmu.2026.1845691 (PMC13423976; doi:10.3389/fimmu.2026.1845691)
Supplement: Supplementary file 2 [file DataSheet2.docx]

**Supplementary Information**


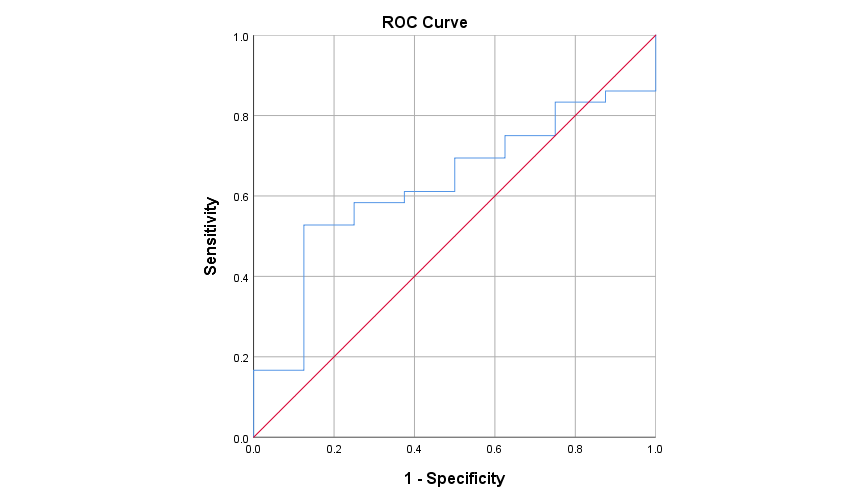


**Supplementary Figure 1.** ROC curve of the density of CD68⁺CD163⁺ M2-like TAMs in the tumor area for PFS status.


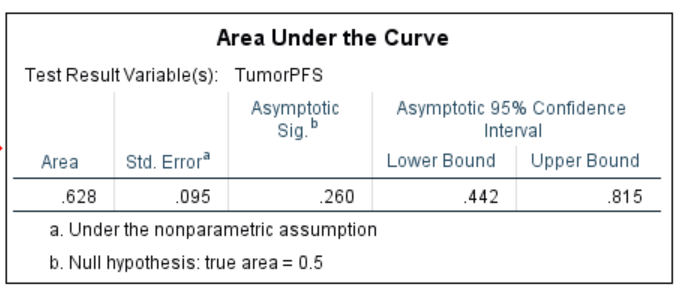


**Supplementary Figure 2.** Statistical Analysis of ROC Curves for PFS status.

Area under the curve (AUC): 0.628, 95% confidence interval (CI): 0.442-0.815, P = 0.260.


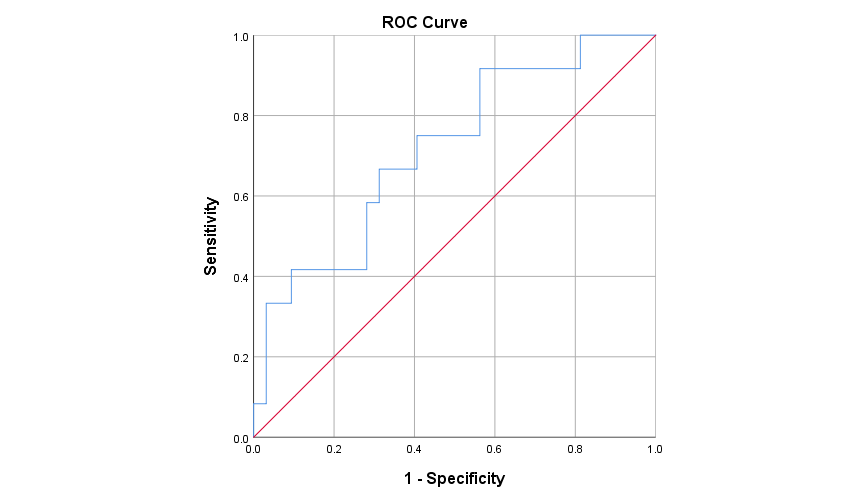


**Supplementary Figure 3.** ROC curve of the density of CD68⁺CD163⁺ M2-like TAMs in the tumor area for OS status.


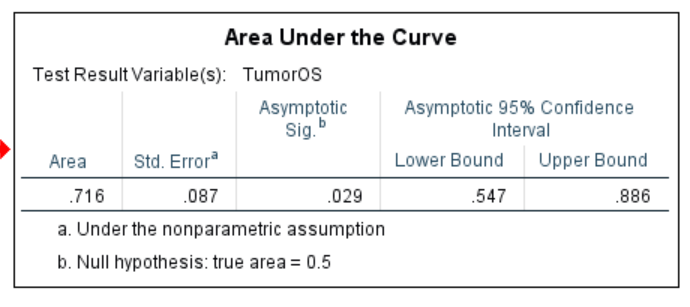


**Supplementary Figure 4.** Statistical Analysis of ROC Curves for OS status.

AUC: 0.716, 95% CI: 0.547-0.886, *p = 0.029.


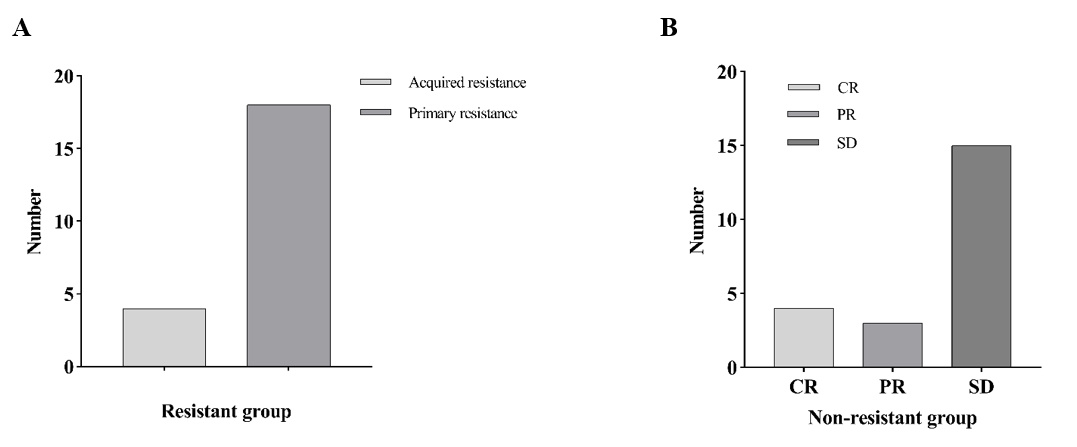


**Supplementary** **Figure 5.** Classification into resistant and non‑resistant groups.

(A) Resistant groups, the majority (18 cases, 81%) were classified as primarily refractory or experiencing early progression, while a small subset (4 cases, 19%) fell into the category of acquired resistance. (B) Non‑resistant groups, 15 cases achieved stable disease (SD), 4 cases achieved complete response (CR), and 3 cases achieved partial response (PR).


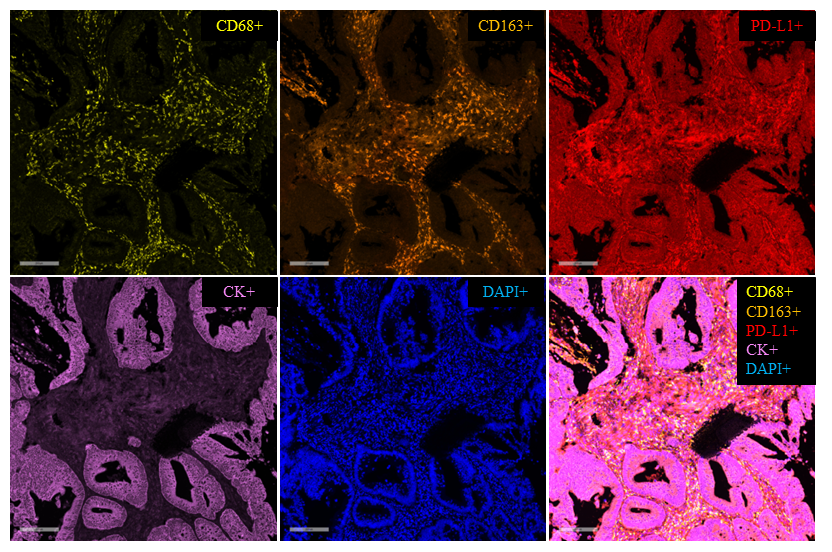
**Supplementary Figure 6.** Composite multiplex immunofluorescence image of the resistant group. Scale bars: 200 μm. The colors indicate positive cells expressing specific markers in the tissue: yellow (CD68), orange (CD163), red (PD‑L1), purple (CK), and blue (DAPI for nuclei).


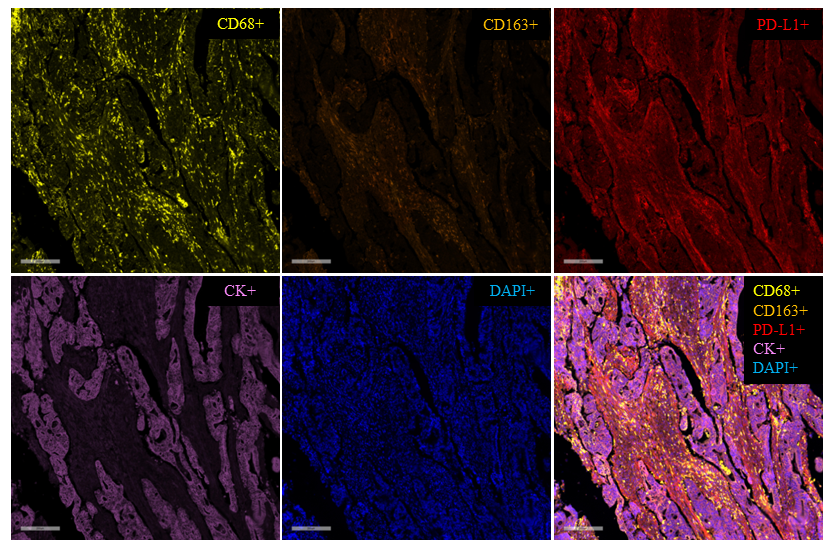


**Supplementary Figure 7.** Composite multiplex immunofluorescence image of Non‑resistant groups. Scale bars: 200 μm. The colors indicate positive cells expressing specific markers in the tissue: yellow (CD68), orange (CD163), red (PD‑L1), purple (CK), and blue (DAPI for nuclei).
